# Changing face of pharmacology practicals for medical undergraduates

**DOI:** 10.4103/0253-7613.56062

**Published:** 2009-08

**Authors:** Mira Desai

**Affiliations:** Indian Journal of Pharmacology, Professor of Pharmacology, B. J. Medical College, Ahmedabad-380016, Gujarat, India. E-mail: desaimirak@yahoo.co.in

A curriculum is a vision and road map to meet the academic objectives. The undergraduate pharmacology curriculum has always been a topic of intense debate. It has been generally felt that pharmacology course in medical schools has failed to keep pace with the rapid changes and requirements of clinical practice.[1] Traditionally, it has focused more on factual information with little or no emphasis on clinical and applied aspects. Dispensing pharmacy and experimental pharmacology has remained the cornerstone of conventional pharmacology practical exercises. Clinical utility and relevance of these practical exercises have always been questioned and criticized.[2] The year 1997 was a turning point when Medical Council of India (MCI) spelt out the objectives for different disciplines.[3] For pharmacology these were broad based ‘to inculcate a rational and scientific basis of therapeutics’, although intricate details were missing. The lack of clarity possibly failed to initiate the task and so the desired impact was not seen. Subsequently, attempts were made by a few medical colleges to introduce practical exercises on drug related clinical problems.[4] With the restrictions on the use of animals, computer assisted learning (CAL) was also suggested to demonstrate the effects of drugs on living tissues and animals.[5] However, it is felt that undergraduate pharmacology practicals have been no more than demonstrations and none of them actually involve the students in the learning process. One always wished to have a module that could take care of all the requirements.

A model undergraduate curriculum was formulated and proposed to MCI in 2007.[6] Meanwhile, another curriculum was suggested by Directorate General of Health Services, Government of India (2008), that demonstrated a clear emphasis on clinical pharmacology. It has been proposed to teach essential skills that will help the students select the medicines safely and effectively throughout their professional life. The curriculum also highlights the therapeutics of some common diseases as a key learning objective. With changing times, topics like clinical trials, ethics, informed consent, use of drugs in special situations (pregnancy, elderly, liver and kidney diseases etc) have also become a part of essential skills. A few medical colleges (including mine) have already incorporated the clinical pharmacology exercises without any radical change in the curriculum. However, a consistent teaching programme does not exist in medical colleges in India[6] and the confusion between conventional to contemporary still prevails.

The questions is, ‘Why we have not been able to reform and revise practical pharmacology teaching?’ Let us introspect the issue with various perspectives. Any change in the existing system is likely to encounter the resistance and challenges at multiple levels. Although not difficult, the reforms need a lot of academic, administrative, financial and logistics support and adjustments. It is easy to delete irrelevant and obsolete practical exercises. However, it is difficult to substitute them with equally relevant, meaningful and feasible exercises that can be implemented within the existing framework without disturbing the teaching and evaluation programme. Now that the clinical pharmacology has been specified in more than one revised curricula, each exercise needs to be designed with definite objectives, materials required, textual information, lesson plan (how the session will be conducted in the given time slot), expectation from the students, checklist (if any), assignments and evaluation plan. Perhaps a practical manual for students and teachers will also be of great help in this regard.

Another constraint is getting approval from the affiliated university. This is likely to be important particularly when there are several colleges under the same university. This would require co-ordination among different institutions. Additionally, some of the exercises (e.g. sources of drug information and P-drugs) may require permission to use textbooks, reference books, ***MIMS*** and ***IDR*** during the practical sessions and examinations. CAL, intravenous drip setting and rate calculation also require special arrangements in the laboratory and procurement of material. The cost constraints and wastage may become a matter of concern for many departments. Further, evaluation of communication skills and CAL may not be feasible in case of large number of students. Since examination is the major drive to learn, lack of assessment may fail to change the behavior and attitude in the learner.

Eventually, what really matters is the quality of therapeutic reasoning and prescribing skills. Currently, these skills are inadequate. The prescription writing exercises are no more than a recall test and they hardly ensure the art of scientific prescribing. Although the clinical pharmacology exercises sensitize the students for a rational use of drugs, it is not known to what extent they improve the prescribing skill. Unfortunately, these skills are not reinforced during clinical terms and so their practical application remains incomplete. Perhaps an inbuilt deficiency of integrated teaching across the medical course may also interfere in the achievement of desired objectives. Such defects shall also have to be corrected along with other structural changes to meet the expected goals.

Simultaneously reforms will also be required in other subjects of medical curriculum to ensure an acceptable academic outcome. Undertaking educational research to find out the success of these interventions will provide a great help in this regard. However, all this requires a team of dedicated teachers with a drive to work hard and get trained in a new pattern of teaching and evaluation. We all need to be prepared for this. The academic and professional satisfaction associated with this endeavor is worth all the difficulties.
